# Radicular Cyst: A Cystic Lesion Involving the Hard Palate

**DOI:** 10.7759/cureus.47030

**Published:** 2023-10-14

**Authors:** Nishant Rathi, Amit Reche, Sakshi Agrawal

**Affiliations:** 1 Public Health Dentistry, Sharad Pawar Dental College, Datta Meghe Institute of Higher Education & Research, Wardha, IND

**Keywords:** fibro-osseous lesion, cyst enucleation, curretage, odontogenic tumor/cyst, radicular cyst

## Abstract

A radicular cyst, also known as a periapical cyst or root end cyst, is a type of odontogenic cyst that is typically associated with permanent teeth. The radicular cyst usually is associated with maxillary central incisors followed by mandibular first molars. It occurs as a result of bacterial infection and pulpal necrosis which leads to inflammatory stimulation of the epithelial cell rests of Malassez along the periodontal ligament area of the tooth. Most cases of the radicular cyst are asymptomatic and they are diagnosed accidentally during routine radiographic examination. This article presents a case report of a 42-year-old male with an apical periodontal cyst associated with the maxillary anterior region. Early diagnosis and treatment planning is necessary. This article signifies the role of the surgeon in the early diagnosis and treatment plan of the cyst.

## Introduction

In the intricate landscape of oral and maxillofacial pathology, radicular cysts stand as intriguing enigmas. These benign yet potentially destructive lesions have long perplexed dental and medical professionals alike, demanding a deeper understanding to ensure timely diagnosis and effective management [1]. As we embark on this journey through the fascinating realm of radicular cysts, we will delve into their origins, manifestations, diagnostic methodologies, and treatment modalities [2]. Radicular cysts, also referred to as periapical cysts, emerge from a complex interplay of factors within the dental pulp [3]. These cysts have the potential to wreak havoc on the oral cavity if left unchecked, making them a topic of great significance within the fields of dentistry and oral surgery. Understanding the intricacies of radicular cysts is crucial not only for dental practitioners but also for anyone concerned with oral health and well-being [4]. The goal of this article is to increase awareness regarding radicular cysts.

## Case presentation

**Figure 1:**
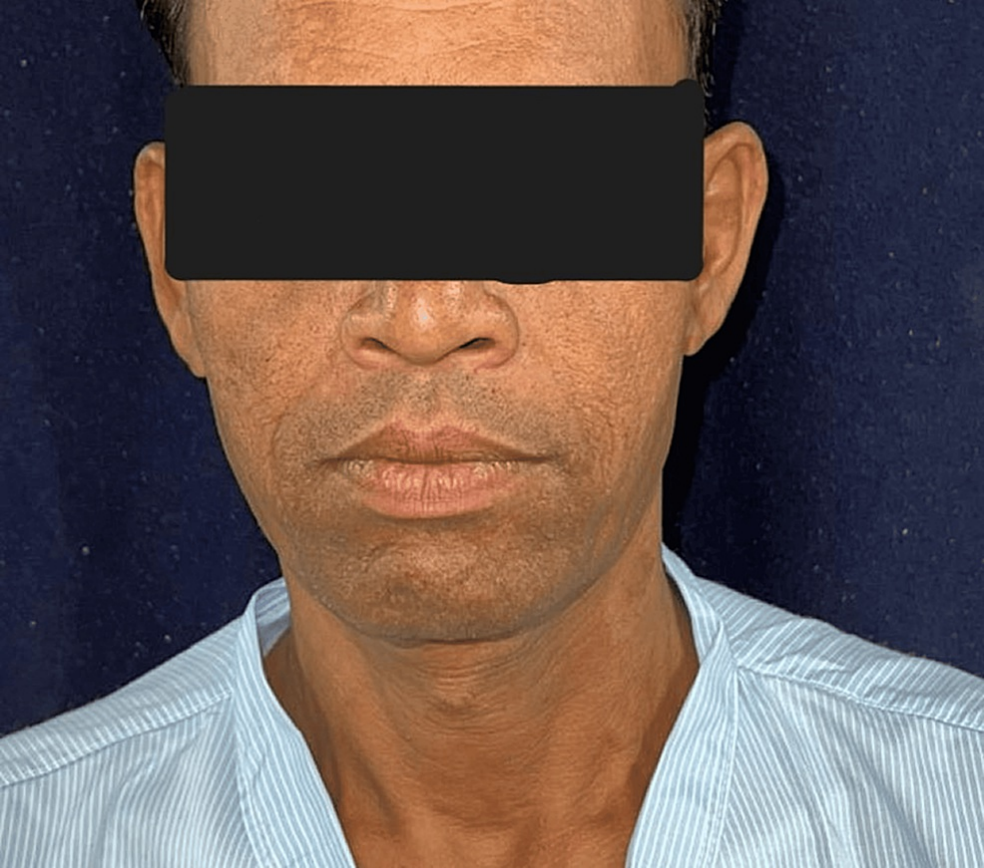
Extraoral examination of the patient.

A 42-year-old patient visited our outpatient department with a one-month history of pain in the upper left anterior jaw, accompanied by swelling for the past 10 days. The patient had previously applied ayurvedic medication and experienced bleeding and pus discharge for about a month. The pain was sharp and intermittent, worsened during chewing, and improved with medications. The patient also reported a history of fever, paresthesia in the area, and discomfort while bending for the past eight days. Over time, this pain worsened and led to the development of swelling in the upper left molar region, which has been progressively increasing in size. The swelling in the upper left molar region gradually increased and reached the present size. On extraoral examination, no facial asymmetry was seen (Figure 1).

**Figure 2:**
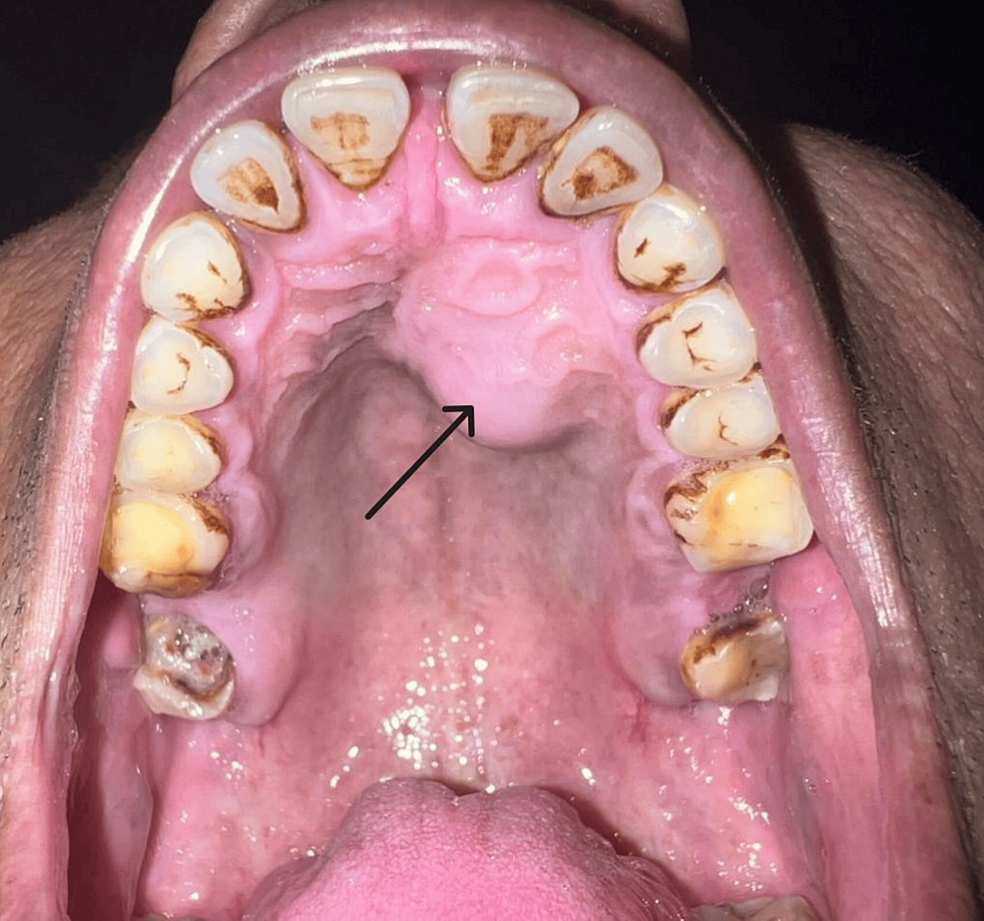
Intra-oral examination showing swelling on the hard palate.

 On intraoral examination, a single swelling was seen in the upper left anterior jaw region associated with 22-24, measuring approximately 2.5 x 2 cm. The shape was roughly oval with a smooth surface, diffused margins, soft consistency, and tenderness was absent (Figure 2). On palpation temperature was raised, the swelling was fixed to the underlying structure and its consistency was soft.

**Figure 3:**
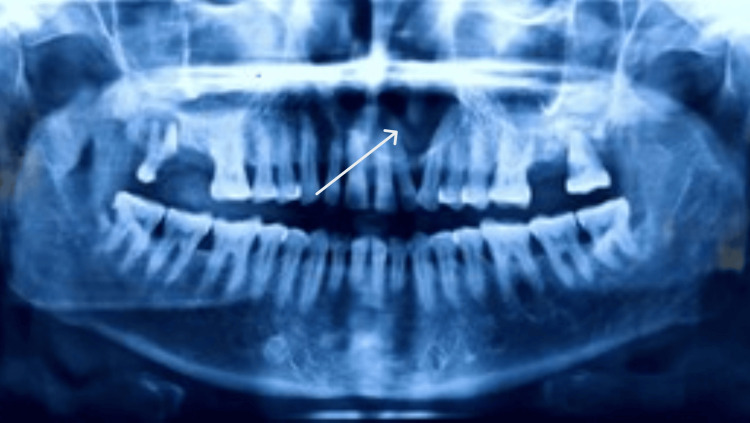
Orthopantomogram (OPG)

An orthopantomogram (OPG) revealed a well-defined radiolucency in the periapical region of 22-24 measuring approximately 2.5 x 2 cm; it was roughly oval in shape and had well-corticated borders (Figure 3).

Based on the patient's history, clinical examination, and radiographic examination, a provisional diagnosis of radicular cyst and acute exacerbation of chronic periapical abscess at 22-24 was made. According to pulpal diagnosis, the tooth was nonvital, and according to periapical diagnosis, displayed acute exacerbation of a chronic periapical abscess. Based on the surgeons' point of view and assessment of all the necessary findings, a clinical diagnosis of radicular cyst with 22-24 was confirmed. It was decided to surgically incise the lesion under local anesthesia along with enucleation and curettage.

## Discussion

Radicular cysts are more common in males than in females [5]. They tend to occur more frequently in the maxillary dentition than in the mandibular dentition. These cysts are the most common type that develops in the jaw and are derived from cell rests of Malassez located around the roots of teeth. Due to their rare occurrence, they often go unnoticed [6,7]. However, they are expected to resolve once the primary tooth falls off or is extracted and therefore are often left untreated. Radicular cysts are usually asymptomatic unless they become secondarily infected [8]. On radiographs, radicular cysts appear radiolucent and typically have well-corticated borders. In rare cases, they may exceed 1 cm in size and show buccal or lingual cortical plate expansion, which can lead to bone thinning around the affected tooth [9]. In young adults, cyst formation can result in bone resorption, delayed eruption, malposition, enamel abnormalities, or damage to the developing permanent successors. However, postsurgical osseous lesions always resolve well because they have a high predisposition for bone regeneration. Surgical treatment of apical periodontal cysts is almost always enucleation, and it is mostly recommended that the permanent teeth associated with the lesion should be preserved, whereas the preservation of deciduous teeth may vary according to the situation. Surgical treatment of apical periodontal cysts typically involves enucleation. It’s generally recommended to preserve permanent teeth associated with the lesion, while the approach may vary for deciduous teeth depending on the situation. Other commonly performed surgeries include marsupialization (Partsch method), enucleation with primary packing, or marsupialization followed by enucleation (Waldron’s technique) [10,11].

## Conclusions

In conclusion, radicular cysts are common yet often unnoticed oral conditions, potentially affecting both primary and permanent dentition. Close post-surgery follow-up is advisable, for bone regeneration purposes. However, to date, this patient is not ready for further follow-up.
